# Unusual presentation of multiple nerve entrapment: a case report

**DOI:** 10.11604/pamj.2014.19.283.5665

**Published:** 2014-11-15

**Authors:** Veli Citisli, Murat Kocaoglu, Selcuk Göcmen, Mustafa Korucu

**Affiliations:** 1 Department of Neurosurgery, Medical Faculty of University of Pamukkale, Denizli, Turkey; 2 Department of Neurosurgery, Private Denizli Surgical Hospital, Denizli, Turkey

**Keywords:** Cubital tunnel syndrome, ulnar nerve, electromyography

## Abstract

Cubital tunnel syndrome is the most common form of ulnar nerve entrapment and the second most common entrapment neuropathy of the upper extremity after carpal tunnel syndrome. However, bilateral compressive ulnar neuropathy is a rare condition. Electro diagnostic studies are a valid and reliable means of confirming the diagnosis.

## Introduction

Cubital tunnel syndrome is the most common form of ulnar nerve entrapment and the second most common entrapment neuropathy of the upper extremity following carpal tunnel syndrome [1–5]. Ulnar nerve can be entrapped at multiple sites of the upper extremity, from the cervical nerve roots C8/T1 and brachial plexus to more distal sites at the elbow, for earm and wrist. Elbow entrapment is seen most commonly and has been referred to as the tardy ulnar nerve palsy in the past. Entrapment of the ulnar nerve has been recognized for more than 100 years [1]; however, the term “cubital tunnel syndrome” is the term introduced by Feindel and Stratford in 1958 because of its similarity to carpal tunnel syndrome [4]. We presented an unusual case with multiple nerve entrapment.

## Patient and observation

**Figure 1:**
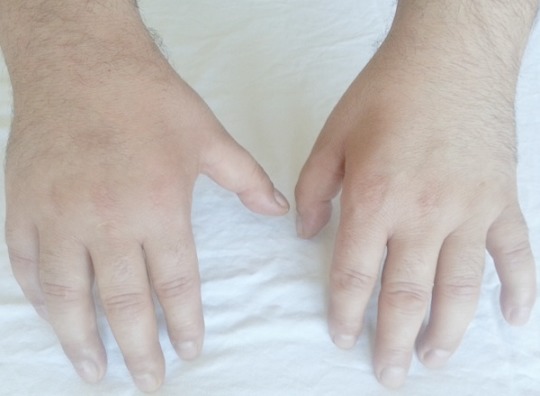
Before the operation, patient's hands

A 38 year old man was examined for bilateral paresthesias over the small and ring fingers. He also reported progressive clumsiness and loss of dexterity in both hands (Figure 1). He had no significant medical history such as rheumatologic and endocrine disorders, or hereditary neuropathy and no history of previous trauma injury. He had been a cashier for 2 month. Neurological examination revealed positive Tinel signs and diminished sensation both fourth and fifth digits and hypotenar eminence at monoflament testing. The vibration test of discrimination was also positive in the same territory. This case was clinically interpreted as bilateral ulnar nerve entrapment and left cubital tunnel syndrome.

**Figure 2:**
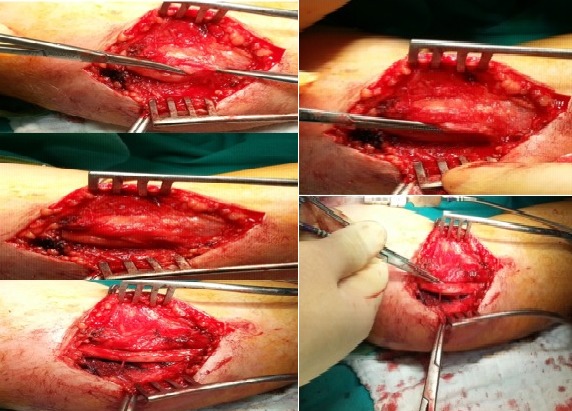
At operation, the left ulnar nerve was compressed by the adhessive bant and muscle

**Figure 3:**
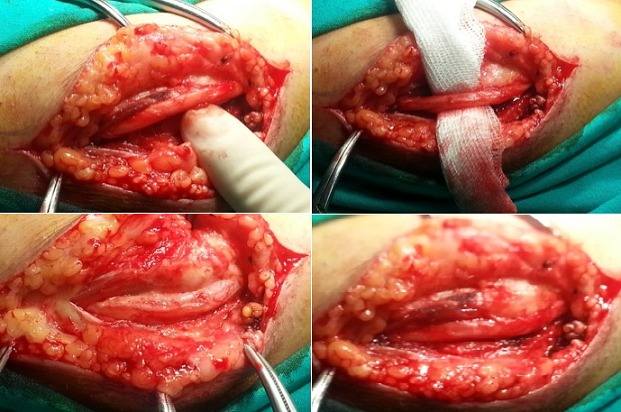
At operation, the right ulnar nerve was compressed by the adhessive bant and muscle

The electromyography (EMG), and nerve conduction studies revealed the focal slowing of bilaterally ulnar nerve and left median nerve. The patient was diagnosed with severe work-related bilateral cubital tunnel syndrome and left carpal tunnel syndrome. The first therapeutic attitude was to change the patient′s way to of working. He was advised to avoid resting the elbows on hand surfaces and he was given an elbow pad to wear over the medial aspect of the elbow to protect the ulnar nerve from direct pressure of trauma. Anti-inflammatory drugs were administered for 3 weeks together with gastric protection. However, the complaints of the patient weren't recovered. Finally, the patient underwent surgery. Primarily simple decompression of the ulnar nerve employed the use of a longitudinal incision approximately 6 to 8 cm long just anterior to the medial epicondyle at the elbow. The medial epicondyle was exposed, allowing identification of the ulnar nerve proximally. The nerve was released proximally as it passed through the medial intermuscular septum. A portion of the medial intermuscular septum was released from its attachment to the medial epicondyle to prevent kinking or compression of the ulnar nerve in elbow flexion. The cubital tunnel retinaculum and flexor carpi ulnaris aponeurosis was divided, which allowed for simple decompression ‘release’ of the ulnar nerve at the elbow. There is commonly a second constricting fascial band deep within the substance of the flexor carpi ulnaris,1 to 2 cm distal to the proximal fibrous arcade that was released. Next, the portion of the ulnar nerve under the medial epicondyle was compressed and was released by dissecting it free from the overlying aponeurosis (Figure 2, Figure 3). Figure 1: showing muscle atrophy between thumb and forefinger and in the late period, the little finger and ring finger bent into palm constantly.

## Discussion

There are 4 sites where the ulnar nerve is frequently vulnerable to compression. These include 1. The arcade of struthers (medial intermuscular septum), 2. the ulnar groove, 3. The humeroulnar arcade (or cubital tunnel), and 4. the exit point between the 2 heads of flexor carpi ulnaris. From these locations, the most common are lesions at the ulnar groove and humeroulnar arcade [1, 3]. One of the most common etiological factors in ulnar neuropathy at the elbow involves compression of the nerve due to entrapment. The most proximal site of compression around the elbow involves the arcade of Struthers [4]. Various reasons exist for lesions occurring to the ulnar nerve within the ulnar groove and cubital tunnel including external trauma, pressure, bony or scar impingement, irregularities in muscles, congenital abnormalities such as combinations of cubitus valgus and anterior dislocation of the head of the radius, and sometimes soft tissue mass lesions, repetitive over head activities, traction, metabolic disorders, mechanical factors (such as stretching of, friction on, or compression of the ulnar nerve), and occupational factors [1, 2]. In addition, the summation of multiple, repetitive episodes of microtrauma can also lead to fibrosis and nerve constriction. This etiological factor in ulnar neuropathy has been evident in baseball pitchers, assembly line workers, violinists, and occupations that involve hammering, shoveling, and/or lifting. However, Entrapment syndromes involving multiple peripheral nerves are common in hereditary neuropathy with liability to pressure palsies and are associated with a number of other conditions, including rheumatic and metabolic diseases, in particular diabetes and connective tissue disorders [6]. The ulnar nerve is vulnerable to trauma in contact works such as cashier, because it lies behind the medial epicondyle an is superficial in to the olecranon fossa as it enters the cubital tunnel [2, 7]. Repetitive elbow flexion causes the ulnar nerve to be stretched, which compresses the nerve with in the cubital tunnel [5, 7, 8]. The many compromising positions the elbow is placed while working as a cashier could easily be defined as excessive or repetitive. However, we were unable to identify any reports of bilateral cubital tunnel syndrome and unilateral carpal tunnel syndrome involving cashier worker. Compression of the ulnar nerve under the edge of the flexor carpi ulnaris aponeurosis can also result in ulnar nerve neuropathies. The aponeurosis can be thick and fibrotic, and by flexing the elbow, the aponeurosis tightens, further constricting the cubital tunnel space. This, in turn, compresses the ulnar nerve. The entrapment site is located 5 to 7 cm distal to the medial epicondyle [1], Finally, flexion of the elbow for prolonged periods can also lead to ulnar neuropathies at the elbow (eg, immobilization of an arm following fracture, or dislocation of upper arm or shoulder). Prolonged elbow flexion puts a tremendous stretch on the nerve and simultaneously changes the diameter of the nerve, compressing it [1].

Symptoms of cubital tunnel may start insidiously or acutely, the latter being more common with trauma. The clinical symptoms relate to the mixed sensory and motor neural fibers of this nerve in early disease. The patient generally has paresthesias radiating distally in the hand over the fifth finger and ulnar aspect of the fourth finger [1, 4]. Symptoms progress from mild intermittent numbness induced with elbow flexion to constant anesthesia. Pain and tenderness over the medial epicondyle and cubital tunnel may be present with proximal or distal extension of the elbow. Weakness of ulnar nerve-innervated intrinsic hand muscles can also be seen at this time. This symptom observed alone may be more likely because of the improper functioning of the C8 and T1 nerve roots. The flexor carpi ulnaris and the ulnar half of the flexor digitorum profundus are usually not affected. Weakness starts with clumsiness and loss of dexterity of the hand, with progression to weakness of grip and pinch (Froment sign). Atrophy of the intrinsic hand muscles, as well as “clawing” of the fourth and fifth fingers is an indication of advanced motor loss. This latter classic sign, better known as “claw hand” “benediction posture” or “main en griffe” is produced by the hyperextension of the metacarpophalangeal joints of the fourth and fifth digits and flexion of the interphalangeal joints, because their interossei and lumbrical muscles are paralyzed. Consequently, the patient cannot flex the metacarpophalangeal joints or extend the interphalangeal joints. Because of this phenomenon, patients with ulnar nerve injury are likely to have difficulty in making a fist [1].

Once the location and severity of the neuropathy has been correctly determined, a treatment protocol can be established. In the cases where the symptoms are mild to moderate, conservative treatment can be administered [1]. In treating cubital tunnel syndrome, the goal of conservative treatment is 2-fold: to eliminate, or reduce, the frequency of external compression on the nerve and to minimize flexion at the elbow joint. Night splints are an excellent method of minimizing elbow flexion during sleep. Night splinting is important because many individuals may inadvertently flex their elbows while sleeping [1]. Only in severe cases of cubital tunnel syndrome is daytime splinting advised. The patient is usually fitted with a thermosensitive molded plastic splint that will ensure elbow immobilization during the day. If there is a continual ulnar nerve instability, distal neural symptoms, and/or pain that limits performance despite a comprehensive nonoperative rehabilitation program, the patient is most likely a candidate for surgical intervention. Many diagnostic studies are helpful to validate the suspected diagnosis and can also rule out certain causes of cubital tunnel compression. Neurophysiological studies are helpful in establishing diagnosis and should be done if surgery is planned, in order to document preoperative baseline. Ulnar nerve velocity of <50 m/s at the elbow is considered positive for cubital tunnel syndrome [4]. As far as we know, both this is the first case of bilateral compression neuropathies described in a cashier and the association of bilateral cubital tunnel syndrome and left carpal tunnel syndrome hasn't been described in cashier exposed to biomechanical risk factors. In so much that, the biomechanical exposure involved in cashier working has not been previously analysed.

## Conclusion

In conclusion, a careful examination of the patient with peripheral nerve entrapment is critical. Multiple nerve entrapment should be investigated differential diagnosis of compressive neuropathies. Fast diagnosis and immediate treatment are mandatory to regain best possible recovery.
